# “I Don’t Even Want to Come Out”: the Suppressed Voices of Our Future and Opening the Lid on Sexual and Gender Minority Youth Workplace Discrimination in Europe: a Qualitative Study

**DOI:** 10.1007/s13178-021-00644-0

**Published:** 2021-09-30

**Authors:** Radhika Seiler-Ramadas, Lovro Markovic, Chase Staras, Laura Llop Medina, Jelena Perak, Christina Carmichael, Matej Horvat, Mario Bajkusa, Sladjana Baros, Lee Smith, Daragh T. McDermott, Igor Grabovac

**Affiliations:** 1 Department of Social and Preventive Medicine, Center for Public Health, Medical University of Vienna, Vienna, Austria; 2 School of Psychology & Sport Science, Anglia Ruskin University, Cambridge, UK; 3 Polibienestar Research Institute, University of Valencia, Valencia, Spain; 4 Forum for Freedom in Education, Zagreb, Croatia; 5 Centre for Health, Performance and Wellbeing, Anglia Ruskin University, Cambridge, UK; 6 Faculty of Law, Comenius University, Bratislava, Slovakia; 7 Centre for Disease Control and Prevention, Institute of Public Health of Serbia “Dr. Milan JovanovicBatut”, Belgrade, Serbia; 8 NTU Psychology, School of Social Sciences, Nottingham Trent University, Nottingham, UK

**Keywords:** Workplace discrimination, LGBT youth, Sexual minority, Mental health, Anti-discrimination policy

## Abstract

**Introduction:**

In Europe, young sexual and gender minority (SGM) people continue to face discrimination in the labour sector despite advances in legislation towards their acceptance and equal treatment. Non-discrimination policy strategies helping SGM individuals are not equally enforced in all contexts, making it difficult for many SGM individuals to disclose their identity, hence undermining their health and well-being.

**Methods:**

Qualitative semi-structured interviews were conducted between October 2020 and February 2021 with 55 SGM youth (18–27 years) having work experience from Austria, Croatia, Serbia, Slovakia, Spain and the UK.

**Results:**

From the analysis, three overarching themes were significant: (1) societal discrimination played a major role in sociocultural factors and policy considerations, (2) workplace discrimination had distinct factors and impacts on SGM individuals and (3) SGM inclusion should use strategies to ensure workplace diversity and equality.

**Conclusions:**

SGM individuals from contexts of poor acceptance tended to hide their identity in the workplace, while transgender and non-binary individuals were prone to experience force-disclosure and discrimination in all aspects of employment. There is a lack of resolute reaction from policy makers in managing problems faced by SGM people in workplaces. New laws improving the status of SGM people need to be further adopted, staff training should be implemented, and managers are crucial in achieving an inclusive climate in the workplace.

Policy Implications

It is essential to implement policies on how to effectively handle problems faced by sexual and gender minority people in the workplace.

## Introduction

Individuals of sexual and gender minority (SGM) experience some of the most systemic and persistent forms of discrimination in comparison to other marginalised populations in modern societies and contexts (Ozeren, 2014). Despite advances in legislation towards the acceptance and equal treatment of SGM individuals, they are still at greater risk of unfair treatment, systematic oppression and even violence in large part due to widespread social stigma (Webster et al., 2018). In Europe, young SGM people continue to face discrimination in all walks of life. This is often experienced in the labour sector, where SGM individuals are likely to hide their identity or in more oppressive nations endure harassment for fear of losing their job (Takács, 2006). Research focusing on the SGM community found that 20 to 50% of respondents across European countries felt discriminated during their job search and/or at work (Lloren & Parini, 2017). Young people are particularly vulnerable to workplace discrimination, as from the point of puberty they are faced with issues including possible non-acceptance by family or social networks and bullying and harassment at school, at the same time as they are transitioning into adulthood (Takács, 2006). Discrimination denies SGM people equal access and treatment in workplaces, leading to the exacerbation of poor mental, physical and social health (Takács, 2006). Hence, young SGM workers often suffer under intense social pressures and workplace bullying in comparison to other individuals within the working environment (Branch et al., 2012; Lamberts & Pauwels, 2006).

Despite calls for SGM workplace discrimination to be central to research on human resource management, very little has been done to voice the experiences of SGM individuals in the workforce (Ozturk & Tatli, 2016). As policy strategies are not equally available in all contexts, there is a need not just for the acceptance and understanding of SGM people at the individual level, but also for companies, society and the political system to get involved and expand their freedoms and labour rights (Mara et al., 2021). Furthermore, many problems faced by SGM individuals originate within frameworks that anti-discrimination policies reinforce. For example, the gender equality, gender management and gender mainstreaming approaches adopted by employers are predominantly hetero- and cisnormative and target stakeholders in a framework of being white, cisgender and heterosexual, hence tending to overlook most problems faced by people from the SGM community (García Johnson & Otto, 2019).

Issues surrounding sexuality and gender minorities in the context of diversity promotion at work is not only an under-researched area but also one of the most challenging to research. This is because SGM people have to play an active role in the “acknowledgement” process through coming out to the researcher and/or to their colleagues, which is difficult to achieve as many SGM individuals may not wish to disclose their identity (Ozeren, 2014; Priola et al., 2014). Self-disclosure also varies with age, religion, geography and job position. Older people, people living in more progressive societal contexts and those who are not influenced by religious beliefs are more inclined to coming out in general (Charoensap-Kelly et al., 2020). Conversely, a lower degree of outness is linked to many external and individual factors of discrimination and preconceptions influencing one’s internalised sexual prejudice (Maciel & Barnett, 2021). While psychological well-being, life satisfaction, self-esteem, resilience and positive work attitudes are higher among those who are publicly committed to their sexual identity, higher levels of chronic stress leading to poorer mental and physical health outcomes, as well as productivity loss, absenteeism and negative job outcomes are common among those who conceal it (DeSouza et al., 2017; Grabovac & Mustajbegović, 2015). Some of the significant factors that are known to be conducive for “outness” in the workplace are an SGM friendly environment, work council protections and a labour management anti-discrimination contract (Markovic et al., 2021).

Recent research also indicates that there are extensive benefits to equal treatment and non-discrimination on labour output, and diversity management in the workplace can have positive consequences for both employees and companies (Badgett et al., 2013; Markovic et al., 2021). According to a statement from a multi-national corporation, an SGM inclusive work ethos is not only the socially right thing to do, but it creates a culture of equality that is crucial to the success of the company and generates confidence, innovation and therefore business growth (Zugelder & Champagne, 2018). Notably, SGM inclusion is appealing to non SGM job seekers, as it reflects on the broader attitudes related to acceptance and diversity within the company (Zugelder & Champagne, 2018). Workplaces further provide the opportunity to educate workers and promote diversity and inclusion, which is especially relevant for younger SGM people entering the workforce (Strunk & Takewell, 2014). Hence, it is especially important to involve young SGM people in exploring the issue of workplace discrimination in order to develop relevant and effective inclusion policies.

Against this background, a group of six partner countries in Europe have been targeting the promotion of an effective implementation of the principle of non-discrimination for SGM youth in the workplace, in the scope of the WE-Project: Promoting Work-Based Equality for LGBT+Q+ Youth, funded by the European Union´s Rights, Equality and Citizenship Programme (GA: 881910). The overall aim of the WE-Project is to investigate the experiences of young SGM people in and around employment, to explore views from stakeholders working with SGM people as well as to gather employment best practices in SGM workplace inclusivity. The goal is to create an online open access platform that will provide educational materials, online courses and a place to present cases of SGM discrimination in the workplace based on our findings. The online platform further strives to gather various solutions and best practice examples of promoting diversity, as well as suggestions on how these may be transferred into other settings on an ongoing basis. The central aims of this part of our study were (1) to explore the individual perspectives of SGM youth regarding their experiences of discrimination in general, and at the workplace, (2) to explore the factors that facilitate or prevent discrimination and (3) to identify strategies that would be more inclusive for young SGM individuals in workplace settings.

## Methods

A qualitative research methodology was applied in our study in order to explore participants’ perspectives and experiences in detail. This approach allows for an in-depth perspective of SGM discrimination as experienced by youth and their opinions on the current status of policies that aim to prevent or deal with SGM discrimination in the workplace.

Our study comprised semi-structured in-depth interviews conducted with SGM youth in each of the six partner countries, namely, Austria, Croatia, Serbia, Slovakia, Spain and the UK. The inclusion criteria were sexual and gender minority persons between 15 and 26 years of age who had any kind of experience in volunteer or paid employment. The key data of our respondents are presented in Table 1.

**Table 1:** Sexual orientation and gender identity of participants, with demographic characteristics

Variable	Countries											
	Austria (*n* = 16)		Croatia (*n* = 8)		Serbia (*n* = 8)		Slovakia (*n* = 7)		Spain (*n* = 7)		UK (*n* = 9)	
	Mean	SD	Mean	SD	Mean	SD	Mean	SD	Mean	SD	Mean	SD
Age	22	2.50	24.38	2.45	23.38	2.26	23.29	3.30	24.14	1.57	22.50	1.41
	***n***	***%***	***n***	***%***	***n***	***%***	***n***	***%***	***n***	***%***	***n***	***%***
Trans & non-binary	5	31.3%	1	12.5%	2	25%	0	0	1	14.3%	2	22.2%
Bisexual	1	6.3%	1	12.5%	2	25%	0	0	2	28.6%	1	11.1%
Gay	7	43.8%	4	50%	0	0	6	85.7%	3	42.9%	2	22.2%
Lesbian	2	12.5%	2	25%	3	37.5%	1	14.3%	1	14.3%	3	33.3%
Intersex	1	6.3%	0	0	0	0	0	0	0	0	0	0
Pansexual female	0	0	0	0	1	12.5%	0	0	0	0	1	11.1%
Student (with current or past employment experience)	11	68.8%	4	50%	4	50%	0	0	2	28.6%	0	0
Full time employed	5	31.3%	3	37.5%	4	50%	7	100%	5	71.4%	9	100%
Unemployed	0	0	1	12.5%	0	0	0	0	0	0	0	0

### Data Collection

Participants from the consortium countries were contacted through various sources including social media channels such as Twitter, Facebook, Instagram and Tik Tok, non-governmental organisations, private sectors and self-help groups. Our team of researchers across the consortium employed snowball sampling and chain sampling strategies by disseminating information on the purpose and goals of the study via our project’s social media accounts as well as through advertisements created specifically for the qualitative study. Information on our interview recruitment was also spread by word of mouth in order to initiate the process of recruitment as soon as the project officially started.

Semi-structured in-depth interviews were conducted in a large qualitative exploratory study on promoting work-based equality for SGM youth. A total of 55 participants took part in the interviews from all the partner countries, of whom 16 were Austrian, 8 were Croatian, 8 were Serbian, 7 were Slovakian, 7 were Spanish and 9 were British. Most participants (98.2%) were involved in part time or full-time employment.

Member countries agreed that online interviews had to be done in most cases where social distancing measures (necessary due to the COVID-19 pandemic) were not possible for interviews that were held in physical presence. Various online interview tools such as Zoom, Webex and MS Teams were used in order to facilitate the face-to-face interviews. The interviews averaged 40 to 60 min and were audio-recorded. A reflective diary was kept by interviewers in order to minimise any bias that may have arisen through researcher’s perspectives.

### Interview Guidelines

The topic guides for the interviews were developed based on the thematic and dynamic interviewing model by Kvale and Brinkmann (Brinkmann & Kvale, 2015). Through the collaboration of our interdisciplinary team with extensive experience in LGBT+Q+ discrimination, a first version of the topics was developed, which was then discussed with our project partners before being finalised into an interview guide for SGM youth participants. This was then translated by our partners into their respective languages and back-translated to check for consistency in meaning.

The interview guide (Table 2) for the youth participants was based on the following questions:

1. How do you perceive discrimination generally/at your workplace, and how are you addressing this discrimination?
2. What are the obstacles that prevent you from addressing discrimination, and the facilitators that would help to address it?
3. How can people be more inclusive of LGBT+Q+ individuals in the workplace setting?

**Table 2:** Interview guide for SGM youth participants

1. How do you perceive discrimination generally/at your workplace, and how are you addressing this discrimination?
What discrimination have you experienced based on your sexuality or gender identity?
How have you perceived/reacted to those instances?
2. What are the obstacles that prevent you from addressing discrimination, and the facilitators that would help to address it?
How have you dealt with discrimination in your job /work experience?
What barriers (emotional, social or structural) may have prevented you from reporting?
What factors could help you to tackle these instances?
3. How can people be more inclusive of LGBT+ individuals in the workplace setting?
What do you think needs improvement in order to eliminate discrimination at work? (What are the structural, social, and /or interpersonal issues that need to be improved)?
What do you think could the government and/or your employer do to make you and the LGBT+Q+ community feel more at ease?

These questions served as a general guideline, and the wording and depth of prompting questions were decided upon by the interviewers during their interview sessions, depending on their judgment on the direction of the interview. This ensured that the interviewees had sufficient freedom to express the issues that were most important for them.

### Ethical Consideration

All participants provided a signed informed consent form as well as their verbal consent prior to participating in the sessions. Participants were pseudonymised with a participant number and national EU coding abbreviation (e.g. AT_1 meaning Austrian participant 1), and employment references were generalised to secure anonymity. The audio files and transcription data were erased by interviewers and/or assistant interviewers and transcribers and kept locked and/or password secured by the respective study teams. The study was approved by the Ethics Committee of the Medical University of Vienna, which served as the lead ethics committee for the project (EKNr:1906/2020). Additionally, each individual member country obtained approval from their designated ethical review board.

### Data Analysis

All interviews, which were carried out by the members of the consortium countries in their respective national languages were then transcribed manually, or with the help of transcription tools such as “otter.ai” and “Amberscript”. The transcriptions were quality checked by the respective research teams before they analysed them with the help of Atlas.ti. (Scientific Software, Berlin; version 8) or Dedoose 8.3.45. The transcripts were analysed for meaning units and encoded following the general approach detailed by Saldaña (Saldaña, 2021). During this process of thematic analysis, the texts are broken down into shorter fragments that could be labelled in one or a few words that are relevant to the research context. The process of deriving the codes was agreed upon collaboratively, but the coding was done individually on a country-by-country basis and checked by the co-ordinating team for inter-coder reliability. The codes were then grouped into categories and overarching themes that were iteratively developed among the qualitative analysts from all member countries. These themes were discussed in each individual country analysis, and respectively exemplified with quotes. The reports and quotes were then translated into English, and were back translated independently in order to verify the meanings behind them.

### Findings

Three main topics emerged from the analysis of the reports. They were namely the *societal discrimination* experienced by participants in which sociocultural factors, and the lack of policy considerations on the handling of SGM discrimination were perceived to play major roles. *Workplace discrimination and its impact* was another theme as participants often highlighted the factors that they perceived were causing discrimination at the workplace, as well as the impact they had on SGM youth. *SGM inclusion policy* was a further topic that emerged through participants’ descriptions of important considerations and strategies that could be used to bring more diversity and SGM equality in workplaces. Further details of the pseudonymised participants and a brief summary of their comments are provided in Table 3 (Appendix).

### Societal Discrimination

#### Sociocultural Factors

A key theme that was commonly mentioned was how SGM discrimination was deeply entrenched in society. Some participants described how society sees them differently, being unable to fit them into a heteronormative culture and tradition where conformism is emphasised, sometimes through religious beliefs where “gay people are seen in a different light” (HR_5). They feel seen as a “threat to the institution of the family” (RS_8) and sometimes even as “having a pathological condition” (RS_5). In some contexts, participants mentioned that the biased views of the government were not allowing for the visibility and acceptance of SGM. One participant was frustrated about the extent to which political members would go to place individuals of SGM in bad light in order to uphold traditional family units:

…because they want to make quick and cheap political points in pointing out our existence and demonize us. (SK_ 7)

Discrimination was pervasive to the extent that participants felt unaccepted by their own families and that they had to struggle with their identity in order to live with them or feel accepted by them. A particularly poignant example was given by one trans participant, who mentioned that they put up with their family not accepting their gender affirmation process in order not to lose them:

*I prefer not to have a family at all, so that I am seen as I am. That’s important to me, I have to - I have to somehow accept that they call me Sara (pseudonym), and just see me as I’m not actually, rather than losing them. (AT_14)*

All participants felt that society lacked knowledge about SGM individuals, and alluded that the education system failed to take the opportunity to raise awareness of SGM and the issues they face. Participants from all participating countries pointed out that SGM people were stereotyped based on their tone of voice, how they dressed, behaved or looked, and that they were quickly categorised and judged unfavourably. Participants across countries felt that experiences in school that related to their gender or sexuality had impacted upon their thoughts and apprehensions at the workplace. Many participants across countries stated that “there was open discrimination at school” (SK_3), that physical and verbal violence as well as harassment often took place, and that traumatic incidents such as physical distancing, verbal bullying or even physical attacks were common. One participant described how three boys “held me down and tried to dunk my head in the toilet” (AT _10) as part of a spate of bullying that was left unaddressed by school authorities. Another reiterated the lack of engagement from teachers even when bullying happened time and again. This participant pointed out that it was difficult for her at the age of 13 to imagine being able to defend herself. She stated:

*I didn’t feel like the teachers took any notice of that. And it was really bad at some point, but they were all just like…..I don’t know if they really didn’t see it or just turned a blind eye…but that can’t be happening. (AT_1)*

#### Policy Considerations

Participants across countries noted that there was a lack of systemic support for the rights of SGM people, which stemmed from weak or ambiguous reaction towards SGM discrimination. Some participants realised how discrimination is being normalised and “accepted although some forms of behaviour are inherently unacceptable” (HR_5). These forms of behaviour relate to the way people are “accustomed” to hearing talk that is prejudiced towards SGM people or experiencing behaviour that indicates that they are unnatural. Hence people perceive this as normal and would not report that they are discriminated because “most people, unfortunately, accept these discriminations as our everyday life*”* (RS_4). Participants were generally aware that norms prohibiting discrimination existed, but many, especially those from Croatia, Serbia and Slovakia, were wary of the legal system, which failed to effectively and convincingly tackle discrimination “because there is no implementation (of the law)” (RS_6). There was a sense of defeat felt among some participants who realised that reporting discrimination was an exhaustive and futile venture with an uncertain outcome. One participant described that her experience of bringing someone to court for discrimination left her feeling doubtful about the process:

..even if someone is going to sue or something like that, it would take years and years… because, now mine has been in court for ten years, and it will not be over so soon. (HR_7)

This experience highlights the lack of clear, direct and resolute reaction from policy and decision makers, who participants felt had the ability to take these situations into account and turn this around but instead continually failed to do so. As one participant explained in continued frustration and exasperation about the general indifference to SGM individuals, “Even politicians don’t comment, so we can continue talking shit (about SGM)” (HR_1). This and other examples added to the general notion that SGM people lacked visibility and acceptance, and gave clear indication that it was necessary to have policies that could change this. Some respondents pointed out that a crucial step towards acceptance of queer people would be to officially recognise the SGM population:

*.... if we are not fully accepted people* (meaning fully recognised by law), *we will fail to reduce discrimination. (SK_5)*

Full recognition, as stated by this participant meant that SGM were allowed the right to cohabit, to inherit from their partners and to acquire medical information; however, the recognition of SGMs was varyingly felt across participating countries. The most prominently observed discrimination across all countries was the lack of legal provisions and protections toward intersex and gender non-binary people. This was notably reflected through overt discriminatory behaviour that often put people of gender minority in a position of disgrace and powerlessness. One such participant felt that gender non-binary people are often faced with having to make a decision of what gender they belong to. They stated that in these situations, “it’s a kind of ‘decide for yourself what are you now’, that a dehumanisation takes place” (AT_6). The dehumanisation process was vividly described by another participant in an example where “the store owner would come in each week and tell me that it was unnatural, disgusting, that I’m a she-male and that I will never be a man” (UK_6). Behaviours like these emphasise the need for a shift towards policies that recognise and support gender minorities. One participant specifically drew an example of how not understanding gender minority issues allows discrimination to happen in that the legal status of gender was seen as more important than how the participant felt about their name:

one teacher insisted on using ‘Miss’ in front of my name. Ahmm….because she said, since it’s not legally… since I’m not legally non-binary um…she has to use the female prefix. (AT_7)

In the above scenario, an institutional policy on how to handle issues faced by gender minority people may have likely prevented this behaviour from happening, and instead would have prompted the teacher to be more aware or empathetic of the participant’s situation. These examples clearly show the need to scrutinise the legal framework on discrimination of SGM people and to implement policies within public institutions that increase the rights and visibility of SGM, as well as to afford better protection for them. Most participants expressed the need for improved and more precise legislation, more legal actions against perpetrators of discrimination and a more efficient system for protection from discrimination in a broader sense.

### Workplace Discrimination and Its Impact

#### Factors of Discrimination in the Workplace

Various factors such as one’s SGM identity, geographical context, coming out and the level of workplace support were highlighted by participants when recounting incidences of workplace discrimination. In general, participants recalled verbal harassment through inappropriate jokes, comments, suggestions, allusions, insolent questions, statements and gossip. Further to these, other homophobic attitudes were displayed, such as sexual harassment and/or social distancing of the LGBT+Q+ individual by not communicating with them or isolating them from activities. Nonetheless, there were differences in the kind of discrimination faced by LGBT+Q+ individuals, and a clear dichotomy was seen. While cis-gendered sexual minorities experienced implicit, subtle forms of discrimination through jokes that were “derogatory, but made to sound humorous” (ES_2) such as “a ‘scissor sister’…. referring to how lesbian women have sex” (UK_7), transgender individuals experienced overt, often dehumanising forms of discrimination including the blatant refusal to hire them:

He (the employer) said “No, I still don’t want to hire you”, but I was like “why?”. He said, “Because you are a transgender person.” But he is gay and the cafe is gay friendly (RS_5)

Furthermore, the degree of discrimination was differently felt in different countries and states, as well as in rural and urban areas. Discrimination was perceived to be more pervasive in Serbia, Slovakia and Croatia where participants mentioned “getting fired because they are LGBT” (RS_4), and that they were forsaking their identity in order to “somehow preserve one’s existence, if nothing else” (HR_3). Some expressed the feeling of being in a hostile working environment if the intention of discrimination was raised, as this participant stated:

*Emotionally I felt very bad, reporting was not possible, discrimination came secretly from the superiors and it was not possible to prove it with evidence, and no one wanted to testify for fear of being fired and being discriminated against. (SK_5)*

Discrimination was still felt among SGM individuals who stood up for themselves, as some examples from Austria, Spain and the UK have shown. While in some cases attitudes of ignorance were faced when an SGM person was refused being addressed by their name: “Oh, we don’t care, we’re gonna call… I’m gonna call them her/she... I don’t really care,” (UK_3), or choice of clothing: “why should you wear shirts as a woman…when there are gender specific clothes” (AT_12). In other instances, participants felt benevolent discrimination such as one example when the employer realised that the participant was gay: “he changed drastically with me. I mean, he was super nice” (ES_1).

Often people in rural areas were not comfortable about addressing discrimination due to the lack of SGM awareness as well as the strong social bond they experienced with people in smaller communities. Since the situation in the rural workplace is a reflection of the situation in that part of society where people are less aware of SGM issues, its consequences could be more strongly felt and discrimination an additionally daunting matter to address. One lesbian participant who kept her identity a secret recalled her experience of feeling extremely uncomfortable having to work with colleagues who “blatantly condemned a lesbian politician for nothing she had done” (AT_5). Another raised the issue that people did not fight discrimination as this behaviour was even less “normalised in smaller areas” (HR_2) and because they were afraid of being laid off or fired from their job, options of which are fewer in smaller cities.

#### Impact of Workplace Discrimination

Participants reported that the impact of workplace discrimination caused mental instability through experiences such as feeling lost, shameful or anxious to varying degrees. While the experience of some participants was that expressing or revealing their sexual and/or gender identity was enough of a risk to cost them a promotion, be downgraded, to not be hired or even to lose their job, others felt that there were no triggers so long as their SGM identity was not revealed. They felt that it was something to be selectively shared, depending on the “level and maturity” (SK_6) of their colleagues. Some expressed that their existential needs were at risk, hence forsaking the need to express their sexual or gender identity in order to adapt themselves to a rigid work environment. One participant described being protective of his sexual identity and avoided coming out for fear of negative outcomes at the workplace:

I don’t even want to come out. (...) it’s just my personal business and no one else’s. I see no reason why I should tell everyone about my orientation. (SK_3)

Other participants of this study described how personal experiences of discrimination led them to alter their subsequent behaviour and the ways they presented themselves at their workplace. One such participant stated that “because of the people who work daytimes, I did take nail polish off, because … I just couldn’t be bothered to have to listen to it (discrimination)” (UK_4).

Where one’s sexual identity was revealed, there were at times harmful consequences. One participant recounted being treated unfairly and gradually being edged out of his workplace when his boss knew about him being gay: “he decided to keep my colleague and let me go, so this is kind of not logical, because I was more involved in the project than he was” (AT_4). Respondents across countries felt that there was a lack of clear public action to discrimination, and that discrimination has been normalised. Under this context there exists a feeling of insecurity in applying oneself to procedures of reporting discrimination, for fear that revealing or accenting their sexual and /or gender identity may have a negative impact on their employment. Hence, participants have the underlying assumption that they could be fired because of their SGM identity, as this participant stated:

how’s it, how are they going to react? You know, are they going to be okay with this? Am I gonna lose my job? (UK_1)

The impact on gender minority respondents was particularly distressing, as they usually found themselves force-disclosing their identity through job interviews and their transitioning process, often with negative outcomes. While one participant mentioned not being hired simply because they were transgender, others mentioned the emotionally challenging circumstances that lay ahead of them. One participant was particularly picked on and criticised for “not being a real man” (AT_12) in the army as he was unable to do the required physical endurance sessions during his transitioning. Another respondent highlighted the exhausting bureaucratic procedures they had to struggle through in order to get their name acknowledged. Respondents across the countries mentioned that they were perceived as difficult for explaining or asserting themselves, that for instance “secretaries are burdened” (AUT_7) by their persistent reminders, giving the impression that a name change is just a cosmetic issue. Participants also commonly highlighted feeling uncomfortable and unsafe when having to go to a gender-specific bathroom, as:

you get stared at no matter which you choose…um…and you don’t wanna intrude in women’s spaces, but then you also don’t feel safe when you go to men’s spaces (AT_6)

Several respondents have also indicated that the perceived discrimination has affected their choice of educational or career choice directions, where for instance some have mentioned that job sectors such as banking, administration and places such as hospitals where hierarchy is entrenched could be particularly discriminatory. On the other hand, participants perceived fashion, creativity and trade-related labour sectors as more favourable:

*A clothing store or anywhere like this is pretty open…In a bank it transmits a lot of insecurity that someone knows my sexual orientation. I see it as quite closed, and ensure that I study business (ES_1)*

### SGM Youth Work Inclusion Policy

There was a general consensus across participants from all countries that in order for young SGM people to feel included in the workplace, acceptance of SGM people and their rights is essential. In order to facilitate this, participants felt a need for dependable legal frameworks and policies applying to the rights of SGM people and clarity of what discrimination involves. Participants felt that the legal system should rightfully apply anti-discrimination laws into reality and effectively resolve cases of discrimination in order to enable SGM people to “effectively report discrimination to someone” (HR_7). Furthermore, participants felt that a general acceptance of queer people, for instance through “the recognition of same sex partnerships… would help to develop an inclusive policy within the employment sector” (SK_2). Also, participants felt that a network of support that includes psychological and legal assistance is crucial. Some respondents specified that sanctions should be applied for violations of legal provisions related to discrimination on the grounds of personal characteristics. Some respondents thought that “it is necessary to further adopt new laws that would improve the status of SGM people, especially for transgender people” (RS_3).

Participants stressed that managers and employers are key actors in initiating and maintaining an inclusive workplace environment. One participant suggested that the onus lies on the employer to ensure that the rights and safety of the employee are secured through enforcing a non-discrimination policy at the workplace:

*All employers should issue a moral code in the workplace, by which they would be bound and which would guarantee everyone their rights and obligations (including) the right to report discrimination without any consequences for the notifier. This should be primarily in all state bodies. (SK_5)*

Respondents reiterated that managers and superiors were role models in mediating problematic situations and complaints, as well as giving strategic guidance, and that good communication between staff is the backbone of resolving problems. Participants suggested that superiors could help to raise awareness on sexuality and gender minorities through knowledge building workshops and training, such as “a training platform where you had videos and quizzes” (UK_9), hence allowing for space, growth and change, which are important factors in promoting an inclusive work environment.

Some participants felt that having SGM people at managerial levels or as contact persons that are “also outed in the work context is definitely an advantage as there is an automatic trust somehow with each other” (AT_5). Participants noted that overt representation of diversity in the workplace like having “a rainbow flag at the entrance” (AT_3) helped to identify the organisation’s commitment to equality and diversity. Furthermore, having a diverse staff community would help to reduce discrimination. One participant described their workplace scenario as one which could be an example of including SGM diversity in the workplace:

*Because I work with like, a lot of like… the age range is very spread. And like, the races of people at work are very spread as well. So I’ve met a very mixed set of people, which I wouldn’t normally meet on a day to day basis. Yeah. So encouraging that diversity to reduce discrimination.. (would be an inclusive strategy) (UK_9)*

Participants across interviews pointed out that employers should allow for the inclusion of gender-neutral or single bathrooms and safe spaces. As one participant stated, “There was a time where I often times did not go to the toilet even though I wanted to, because I didn’t feel safe and there was no neutral option” (AT_6)*.* Participants also stated that staff should know how to address gender diverse individuals by asking for names and pronouns, and that using heteronormative language could appear prejudiced. Respondents also felt that there should be less rigidity about dress codes and nametags, and that SGM people should be given the choice of “what uniforms to wear, or have them unisex, or offer small badges” (UK_4) to ensure dignity and value for the SGM community.

## Discussion

### Societal Discrimination

Our findings show that discrimination operates across multiple levels within society—from the individual, to interpersonal and community levels. Individual attitudes collectively shape community values, which in turn reflect and influence institutional policies and practices (Hatzenbuehler et al., 2020). Consequently, SGM discrimination is deeply entrenched across societies and as such is more difficult to detect and address than other forms of discrimination. More from being just biological traits, gender and sexuality are based on moral and/or religious values that are rooted in national cultures, and are hence less predisposed to negotiation (Priola et al., 2014). In a heteronormative culture and tradition where conformism to such norms is emphasised and policed, it is difficult for SGM people to construct alternative discourses on sexuality and gender differences as the foundations for such discourse are not recognised or accepted (Priola et al., 2014). Large numbers of people think that being homosexual is a sin (Webster et al., 2018). Some countries in Europe such as Serbia experience significant amounts of political struggle to recognise and accept SGM individuals. On the one hand, they are seen by more conservative groups as sinful and an assault on the traditional family unit, but on the other being SGM friendly is a symbol of what it means to be truly European, and some political parties appear to make this outwardly visible, albeit with resistance (Mikuš, 2011; Slootmaeckers et al., 2016).

As family values often stem from religious and cultural values, being open about one’s sexuality or gender nonconformity can be difficult if one fears rejection from home. Fitting in to the familial context is sometimes more important than disclosure, but this depends on how attached one feels to one’s parent or carer. It has been reported that SGM individuals who disclose their sexuality or gender nonconformity usually feel more attached to at least one parent, and are hence more easily able to engage in conversations on homosexuality and gender differences regardless of cultural or religious values (Katz-Wise et al., 2016).

Disclosure of one’s SGM identity is also made extremely difficult through experiences in public schools where most of the bullying and harassment experiences shape the lives of SGM individuals (Dessel & Rodenborg, 2017). It is most often the case that SGM discrimination becomes normalised when instances of discrimination are inadequately addressed within the framework of school, hence making SGM people feel wrong about themselves and discouraging them from reporting discrimination even at the hiring stage (Dessel & Rodenborg, 2017; Vasquez del Aguila & Cantillon, 2010).

From the occurrences described by participants across these European countries, there is a predominant lack of clear, direct and resolute reaction from policy and decision makers, highlighting the need for policies on how to handle the problems faced by sexual and gender minority people in workplaces. In order to facilitate this, the issues and concerns of SGM people need to be fully recognised by law first in order for discrimination to be reduced. One prime example is the recognition of same sex partnerships. It has been reiterated that the lack of legal recognition for same sex partnerships such as in Serbia and Slovakia increases the disadvantages and vulnerabilities of LGBT+Q+ individuals in the workplace (Vasquez del Aguila & Cantillon, 2010). Where policies for SGM people do exist, these are often poorly understood by employers or there is a failure to enact these.

#### Workplace Discrimination

As far as workplace discrimination is concerned, the kind and intensity of discrimination that were observed to occur across the reporting countries differed for SGM individuals depending on (a) their societal context and (b) their sexuality or gender identity.

As observed in our findings, SGM individuals from contexts of poor acceptance such as in Slovakia, Serbia and Croatia, were likely to hide their identity in the workplace, while those from contexts with more formal acceptance were likely to disclose their identity but yet face discriminatory undercurrents. When the societal context is such that the social change towards SGM acceptance, as well as the legislation and organisational policies around SGM discrimination are slow and under-responsive, SGM individuals feel left with little choice but to hide their identity, accept or brush aside experiences of discrimination (Priola et al., 2014). In countries or contexts where SGM people are more formally accepted, as in Austria, Spain and the UK, discrimination is often felt when the individual decides to disclose their identity. In such situations, the discloser may feel that they are within a broader context of support where they are able to confront the harasser or discuss issues with management. Nevertheless, it can be a tiring and stressful endeavour to constantly have to be educating others on discrimination as well as having to resist the pressure to conform. (Mara et al., 2021).

Differences in discrimination based on sexuality or gender were also observed. It was clear that transgender individuals experienced more discrimination than cisgender individuals through their physical appearance, pronoun use or identity changes. Transgender and non-binary individuals were more susceptible to force-disclosure in as early as job interview stages, more prone to demoralising comments such as “you’re not a real man”, and more likely to feel unsafe in situations such as being forced to go to gender specific locker rooms. As such, they experience higher rates of depression and anxiety than cisgender identities (Bauerband et al., 2019; Borgogna et al., 2019; DeSouza et al., 2017).

As can be seen, having to disclose one’s identity or deciding whether to disclose it at work is often a challenging process as the consequences of revealing it may have a negative impact on employment. The feelings of anxiety, shame and fear of losing their job and existence is due to the stigma associated with being an SGM individual (Webster et al., 2018). This then discourages “coming out” and reinforces the invisibility of SGM individuals in the workplace making it difficult to have satisfying careers and achieve professional well-being (Vasquez del Aguila & Cantillon, 2010). Restricting disclosure of one’s sexual identity may give the individual some control over how included they feel in the workplace as they can decide with whom they would feel comfortable talking about themselves. However, concealing one’s identity has been found to negatively affect employees’ feelings of wellbeing and belongingness in the company, while disclosure has been found to be associated with higher job satisfaction and lower job anxiety (DeSouza et al., 2017; Mara et al., 2021)

Participants across countries mentioned their inclination to apply themselves to specific job sectors or educational paths, depending on whether they would face workplace discrimination and are able to justify using coping strategies in their career choice through anticipated discrimination. Although it was more likely that the youth participants on our study had diverse temporary jobs, some clearly stated their preference to avoid formal business environments. Evidence in other contexts have shown that SGM individuals preferred to work in non-profit sectors, and some would apply “occupational sorting” measures, where for instance homosexual men tended to drift towards traditionally female careers that were lower paying, while homosexual women tended to drift toward traditionally male careers that were higher paying (Ng et al., 2012).

#### Work Inclusion Policy

That young SGM individuals should be able to feel that they could safely disclose their sexual or gender identity in workplace settings is what our study strives to accomplish. This point has been raised by studies in the USA, which have shown that SGM employees would feel more supported in environments where they could disclose their sexual and/or gender identities, and that institutions should acknowledge visible and invisible identities of their SGM employees in order to be truly inclusive (Hur, 2020; Sabharwal et al., 2019). While there are unfortunately no specific anti-discrimination laws covering employment in many developing countries, the European legal system recognises the workplace rights of SGM people, and this should provide the necessary impetus for countries across Europe to rightfully apply anti-discrimination laws supporting them (Guasti & Bustikova, 2020; Suriyasarn, 2016). Through this fundamental provision, cases of discrimination would likely be more effectively reported with higher chances of being resolved (Dessel & Rodenborg, 2017). Studies have shown that policies in support of SGM employment non-discrimination had positive effects in terms of increasing equality in pay-scheme, better SGM language inclusion, fewer negative social messages, lower internalised levels of homophobia and higher levels of sexual identity disclosure (Green et al., 2011; Riggle et al., 2010).

As emphasised by our respondents, it is necessary to further adopt new laws that would improve the status of SGM people. For instance, the formal acceptance of LGBT +Q+ employees in the workplace makes them feel less anxious, less threatened, and more comfortable (Hossain et al., 2020). The transgender people in our study experienced more intense extents of discrimination at individual and systemic levels. It has been argued that their being recognised as “individuals who experience gender incongruence”, rather than people who have a “mental disorder” under the *International Classification of Diseases-11* would positively affect how gender identity is viewed by society more broadly, while at the same time ensuring their access to gender-affirming healthcare (Szydlowski, 2016; Thomas et al., 2017). This remains to be seen, but could certainly serve as a backbone to support transgender people in employment, and help them to transition during their period of employment rather than being in and out of employment and/or accepting lower paid jobs, which are experiences they are often faced with (Leppel, 2021; Szydlowski, 2016; Thomas et al., 2017)

Notwithstanding, having policies and practices alone are not sufficient to ensure the absence of discrimination. They need to be consistently enforced and would be done so when the climate of the workplace is conducive (Webster et al., 2018). The fairer and more equitable an organisation is, the better the climate for the employees, and they would be more likely to disclose their sexual identity (DeSouza et al., 2017). Here is where the role of managers and employers as mediators and facilitators of anti-discrimination at the workplace has been underscored by respondents across the participating countries. Contextual support would increase the likelihood of disclosure as there is a reduced perceived risk of stigmatisation and discrimination, lowered psychological strain and increased positive attitudes (Webster et al., 2018).

Our respondents highlighted the significance and importance of education and training. It should duly be noted that the sole promotion of knowledge and awareness without providing the necessary skills is neither sufficient nor effective in trying to introduce real changes in an organization (Vasquez del Aguila & Cantillon, 2010). Instead comprehensive training that emphasizes values such as the meaning behind equality legislation in relation to SGM individuals, the facts and common myths about SGM individuals, and why issues and concerns faced by SGM individuals is a workplace issue for all, are important aspects of work inclusion policies (Vasquez del Aguila & Cantillon, 2010). Hence, when employers engage in educating others, in being proactive about SGM inclusion such as through attending professional conferences on SGM, as well as by serving as a role model, the rights of SGM employees are highly likely to be promoted and protected (Mara et al., 2021).

The acceptance and inclusion of SGM individuals not only increases the pool of talent from which organizations may draw strategic benefits, but also leads to an increase in diversity in different positions and professional teams within the organization (Hossain et al). Having diverse staff also associates with better decision making, efficient problem solving and greater innovation (Hossain et al., 2020).

More specifically, the existing set of studies demonstrates that SGM supportive policies and workplace climates are associated with greater job commitment, improved workplace relationships and increased job satisfaction. Moreover, SGM inclusive settings that advocate fairness, openness, co-operation, support and empowerment would lead to less discrimination as well as improved health outcomes and overall work performance among SGM employees (Badgett et al., 2013; Hur, 2020).

## Limitations

There are various limitations to the study that are important to consider. From the point of view of recruitment, getting young SGM individuals who were below 18 years of age was complex, as parental consent was necessary and for that to be possible, young SGM had the further challenge of having to explain the study to their parent or carer in order to participate. This is a process that not many were willing to go through, as they may have had to disclose their interest and sometimes identity at a time when they were perhaps not ready to do so. In some contexts, such as in Serbia, it was difficult to recruit gay male participants due to the stigmatisation they would be exposed to if they revealed themselves, albeit anonymised, in the interview process. With predominantly snowball sampling, it was difficult to cast a broad net in order to recruit SGM youth from across the sociodemographic, in particular those who may not have had access to smartphones or social media, such as homeless and other marginalised youth. In more SGM accepting contexts, there was likely to be a bias in recruitment towards SGM individuals who were more willing to reveal themselves, to be activists, and to have a degree of assertiveness that may predispose them to navigate and deal with discrimination by themselves.

Another point to consider was the lack of a steady employment situation among many respondents, especially while most were still students. Several of the experiences mentioned were in temporary jobs and in contexts which were not necessarily their ideal places of employment as their main priority may have been to earn money rather than achieve job satisfaction.

## Future Outlook

Presenting the voices of our SGM youth across six European countries, and highlighting the problems they face in workplaces through bringing into view how SGM discrimination is embedded in our society is a definite strength of this paper. To our knowledge, the study is also new in highlighting issues and concerns of SGM people such as how the impact of SGM non-acceptance in law and policies and the unheeded needs of non-binary and transgender individuals prevents workplace inclusion.

It would be important for future studies to include the involvement of professionals and stakeholders such as lawyers, government officials, psychologists, employers and human resource personnel working with SGM individuals, in improving policies on SGM non-discrimination and work inclusion. It would also be informative to further explore the experiences of SGM people in inclusive workplace environments, as well as to compare economic cost evaluations of having an inclusive work environment as opposed to one without any specific inclusive policies or those with high employee turnover rate.

## Conclusion

Our study endeavoured to voice the lived experiences of young SGM individuals from across a European context and illuminate how and why discrimination is happening in workplaces among them. We further wanted to explore the extent to which their rights as societal members are being exercised with the ultimate aim, along with other data gathered in the study, of creating policies of non-discrimination and inclusion of SGM young people in workplaces.

There are extensive benefits to equal treatment and non-discrimination on labour output, and diversity management in the workplace can have positive consequences for both employees and companies. In order for this to happen, there is a crucial need for policies that define discrimination and how to effectively handle the problems faced by sexual and gender minority people. In order to facilitate this, the rights, issues and concerns of SGM people need to be fully recognised by law first in order for discrimination to be reduced.

Our findings have emphasised that sociocultural values shape politics, which in turn reflect on family and self-determined values. Workplace conduct and educational systems are starkly dependent on policies, which as yet broadly lack inclusion of the facts on SGM people as part of our social fabric. Awareness and acceptance of SGM people sets the stage for their inclusion in society, and when this happens, the likelihood of self-understanding and self-disclosure is higher, enabling people of sexual and gender minority to achieve psychological well-being, life satisfaction, self-esteem, resilience and positive work attitudes.

## Appendix

**Table 3:** Sexual and gender minority (SGM) youth participants based per country, based on age, identity, work experience, experiences of personal and workplace discrimination and ideas for SGM workplace inclusive policies. AT = Austria, HR = Croatia, RS = Serbia, SK = Slovakia, ES = Spain, UK = United Kingdom; SOGI = sexual orientation and/or gender identity; T = transgender, B = bisexual, G = gay, L = lesbian, I= intersex, F = female, M = male

**Participant code**	**SOGI**	**Age**	**Current occupation**	**Previous/current work experience**	**Most significant experience of discrimination (personal)**	**Most significant experience of discrimination (workplace)**	**Ideas for inclusivity in workplace or breaking down discrimination in the workplace**
AT_1	T (non-binary, asexual)	22	Student	Part-time retail, service jobs	Being bullied in school with no reaction from staff	Boss refusing to use non-binary reference on nametag	SGM representation in all kinds of media
AT_2	B	19	Student	Voluntary work	None	None, but trans friend was harassed by co-worker for being gender different	Diversity discussions Gender neutral toilets/restrooms
AT_3	G	19	Student	Summer jobs/part-time retail jobs	Disapproving stares/comments when openly affectionate to partner	Sexual life being questioned by a co-worker in an uncomfortable way	Being open about one’s own SOGI, adding SOGI-friendly company labels
AT_4	G	19	Student	Part-time/temporary jobs in service companies	Stressful and hurtful process when parent discovered his sexual orientation	Being unfairly fired despite completing all tasks at work	Rights and consequences of discrimination explicitly written in work contract
AT_5	L	26	Social worker	Social work in shelters	Having to listen to unpleasant statements about lesbians	Having been insulted aggressively for being a lesbian	Having an SGM spokesperson in the work setting
AT_6	T (panssexual)	24	Student	Part-time private, service and retail jobs	Discriminated for being bi-racial and female-presenting	Unfairly blamed by boss that the kid they* babysat was not interacting with them*, when the issue was with the parents	Hasten administrative processes regarding name changes; introduce feminist/queer theory in workshops
AT_7	T (pansexual)	26	Health care worker	Full-time job	Being avoided by people after having disclosed their* non-binary status.	None	Raising awareness of transitioning process
AT_8	G	26	Health care worker	Full-time job	Direct question about sexual preferences	Insensitively mocking imagined sexual appetite, declaring it as a joke	Creating guidelines for further education through workshops and discussions
AT_9	G	24	Student	Various part-time jobs (media, healthcare)	No specific experiences of discrimination	None	Clarifying company’s SGM friendly policy and intolerance towards discrimination; highlight good experiences of SGM people.
AT_10	G	20	Student	Service and healthcare jobs	Three school bullies tried to dunk his head in a toilet	Co-workers refusing to work with him because of his sexual orientation	Staff should be offered courses on recognizing and dealing with discrimination
AT_11	L	21	Student	Volunteer in youth and healthcare groups	Being called a fucking lesbian by a passer-by	Hearing a senior colleague making a homophobic insult directly at an individual	Improved conversational culture and language used about SGM people
AT_12	T (F, in process of transition)	22	Student	Service, government and healthcare jobs	Stared at by someone and asked what they* are	Referred to by a customer as ‘something’ that doesn’t know what the customer wanted	Do not stick to specific heteronormative dress code
AT_13	T (Gay,M)	21	Service staff	Various jobs in the service and entertainment sectors	When a classmate told him that he would shoot a homosexual if he met one.	Colleague was shocked and confused when they had a boyfriend, and not a girlfriend.	Clarify pronouns and how one would like to be called, and state consequences if other staff do not respect that; State man/woman/diverse in job applications
AT_14	I	20	Student	Healthcare internships	Never having come out to family for fear of being rejected	Colleagues insisting on using name assigned at birth, despite name change	Offering courses for awareness of SGM facts, and applying them in practice
AT_15	G	22	Marketing Freelancer	Service and private sector jobs	Being confronted with snide remarks albeit jokes such as ‘those LGBT+ whatever things’	Not being considered for employment because of sexual orientation	Have an internal LGBT+ network Mark Pride Day as a holiday Diminishing hierarchy in interpersonal staff communications
AT_16	G	21	Student	Internship at professional firm, volunteer social work and part-time job in private business	Nothing specific	Nothing specific	Implement specific plan of action against discrimination of LGBT+ people in national legislature
HR_1	B	20	Student	mostly part time jobs (seasonal jobs)	Having issues with family acceptance; enduring SOGI jokes	being silent about sexual and/or gender identity at the university	Talk about sexual and/or gender identity in schools; create special hotline for young people on SOGI topics
HR_2	L	26	Employee	None reported	None reported	None reported	Emphasise person’s competence, not their SOGI; more protections for transgender and non-binary people
HR_3	G	27	Student/employee	Mostly part time jobs and volunteering	None reported	Verbal comments and attacks from colleagues reacting on his SOGI	More role models/coming out among employees; work on acceptance/normalization
HR_4	T (M)	23	Unemployed	Mostly part time jobs (seasonal jobs)	Issues with family acceptance	Verbal attacks; misgendering of pronouns; comments about toilet usage; dissemination of intimate/personal information	None proposed
HR_5	G	25	Employee	None reported	None reported	Exclusion from social activities in the workplace	Anti-stereotype trainings; training on interpersonal relations; provide workshops on SGM
HR_6	G	26	Student	Volunteer/student jobs	Issues with family acceptance	Physical and verbal attacks; exclusion from social events/groups	More courage to self-disclose
HR_7	L	22	Employee	None reported	None reported	Comments about clothing	Educate people about SOGI
HR_8	G	26	Student	Volunteering/student jobs	None reported	None reported	Less emphasis on SOGI, more emphasis on competencies
RS_1	B(F)	23	Student and full-time LGBT activist	Freelancer, barista	Harassment by professor at faculty	Other peoples’ experience: Gay person being fired after complaining of verbal harassment from co-workers; another gay person, gets transferred to lower rank and less paid position after disclosing sexual orientation	Education/trainings for stakeholders—with focus on Human Rights for all, including SGM (to have wider platform) Introducing official policies related to antidiscrimination rules at the workplace
RS_2	B (M)	20	High school student, full-time salesman	Operator	School bullying, based on the word spread by teacher on his SO, but also based on his ethnic identity (Roma);Non-personal experience: violence and attempt of rape of one lesbian, who openly came out.	Denial of labour rights (but not based on SOGI)	Effective internal rules and policies on discrimination; education, and information sharing using different channels
RS_3	T (F, in process of transition)	25	Administrative worker (full time)	Web site developer without formal contract	No specific discrimination experience besides school violence and bullying	Uncomfortable stares from business contacts; Non-personal: Transwoman was refused job in café; Gay friend was bitten by customer after work (physical violence)	Create additional position—communication officer for SGM in different institutions; Advocacy and lobbying for non-discriminatory practices at the WP
RS_4	Pansexual (F)	24	Operator (full time)	Waitress	Public service denial (police refused to provide services after coming out)	Non-personal experience: Transman got fired after coming out as trans.	Sharing information on procedures related to reporting discrimination; education for employers and SGM youth
RS_5	T (M, in process of transition)	26	Salesman (2 part-time jobs)	Restaurant/kitchen (full time jobs)	Institutional discrimination such as: unnecessary demanding of personal, confidential information; refusal to give healthcare services; denial/partial provision of police services. Experiences of physical and family violence	Getting fired after comingout/transitioning; sexual harassment and intimate questioning from employer; open refusal of job because of being trans	Raise awareness of youth and stakeholders on SGM rights and problems; Strengthen SGM youth (self-esteem, self-confidence…) to react on acts of discrimination
RS_6	L	25	Student, internship (part time)	Architect	Social distancing and verbal insults in primary school	Being professionally disqualified by males	Training for secondary school youth in general on professional culture/code, rights etc.; promote + examples of outcomes from reporting discrimination
RS_7	L	24	Student, marketing (full time)	Volunteering in human rights NGOs, various other service jobs	Nothing specific	General homophobic working environment	Online information sharing on LGBT rights, anti-discrimination regulations and procedures; Introducing policies regarding non-discriminatory workplace environment
RS_8	L	20	Babysitting (full time, without contract)	Internship in media and service sectors	Verbal harassment from some teachers at school; being verbally and physically bullied in the school by peers after coming out	Denial of professional education at workplace; got fired due to sexual orientation	Education for youth in secondary school on gender equality, discrimination/procedures, LGBT rights, etc.
SK_1	G	18	Permanent employment	Part-time jobs	Nothing specific	Nothing specific	Public education on SGM facts and concerns; holding discriminator accountable for actions
SK_2	G	25	Entrepreneur	Permanent employment/part time jobs, summer jobs	Nothing specific	Noting specific	Legal recognition of registered partnerships; training on ethics/morality/diversity in the work place; alter the job position of the perpetrators or possibly fire them; SGM education should be mandatory in school for students of all religious backgrounds.
SK_3	G	26	Permanent employment	Summer jobs/part-time jobs locally and abroad	Open discrimination at school	Experiencing disdain from co-workers of both genders, but mainly from men.	Be able to openly discuss discrimination with manager, or HR department; government should support those who are discriminated against
SK_4	G	20	Permanent employment	Part-time/temporary jobs	Fear that things would go wrong if sexual orientation was disclosed	Nothing specific	Social acceptance in general; facilitating open discussion with co-workers; they should be able to ask.
SK_5	G	26	Permanent employment	Permanent employment	None reported	Discrimination that lead to changing jobs	Legal recognition of LGBT people (registered partnerships, inheritance, medical information); adhering to international commitments/obligations of the Slovak Republic; lifting the taboo off the topic so managers can help to fight discrimination.
SK_6	G	22	Permanent employment	Volunteering locally and abroad	Ridiculing SGM people	Ridiculing SGM people	Company’s culture—should support good working environment. Labour unions should be active in this issue.
SK_7	L	26	Permanent employment	Part-time/temporary jobs/summer jobs locally and abroad	Not reported	Gender discrimination was felt, but this reduced after obtaining a university degree and promotion at work.	Managers at work should be responsible for adopting internal standards that prevent discrimination; extremists should stop targeting SGM people despite being trained in labour law; acting as a role model oneself: report discrimination.
ES_1	G	22	Student	Summer jobs/part-time jobs	Insults from University teacher	Harassment	Review code of conduct for companies including sanctions
ES_2	L	23	Working full-time in clothes store	Not disclosed	None reported	Derogatory comments	Compulsory SGM courses in the workplace for all employees
ES_3	G	26	Working full time in a restaurant	Hotels, restaurants and cafes	None reported	Harassment, derogatory comments	Educational courses to normalize different sexual orientation and gender identities
ES_4	B (M)	23	Student	Part-time/temporary jobs	Bullying at school	None as he hides his sexual	Protection mechanisms in contracts
ES_5	B (F)	26	Working full time	Not disclosed	Heteronormative stereotypes because of the way she dressed, which is culturally associated with masculinity	None reported	Educational courses to normalize different sexual orientation and gender identities; educating about non-cis-het sexualities from childhood.
ES_6	G	25	Working full-time	Not disclosed	Homophobic comments at school	None as he considers gay men not to suffer as much discrimination as others within the collective, and considers gay jokes to be harmless.	Educating people from childhood about different sexual orientation and gender identities; improve media visibility.
ES_7	T (M)	24	Working part-time	Summer jobs/part-time jobs	Confusion regarding his documentation, which does not match his appearance and identity	Administrative hurdles	Educating about non-cis-het sexualities from childhood
UK_1	T (Gay, M)	21	Barista	Service industry roles	Misgendering by colleagues and customers	Supervisor that refused to use participant’s chosen name	Gender neutral uniforms
UK_2	L	24	Administrator	Sales assistant	Casual homophobic comments from colleagues	None specified	Inclusive HR policy
UK_3	L	24	Sales assistant/mechanic	Mechanic	Bullying by manager because of being a lesbian	Experiencing workplace bullying	More in-depth employee training and development on SGM communities
UK_4	G	-	Sales assistant	Night Club attendant	Nothing specified	Abuse from customers for their appearance	Mandatory staff training
UK_5	L	21	Lifeguard	N/A	Casual homophobic comments from co-workers	Witness a trans co-worker be removed from a bathroom by a manager	Exploration of diversity matters in team meetings/discussions
UK_6	G	21	Catering Assistant	Retail	Store owner engaging in transphobic behaviour towards them	Reluctance to recognize or promote Pride	Option to pick gender of uniforms
UK_7	B(F)	22	Mechanic	N/A	Casual homophobic comments from colleagues	Witnessing a co-worker being bullied for their non-binary identity	Increasing diversity among the staff base in the organisation
UK_8	T (F)	23	Nursery nurse	N/A	Being misgendered at work	Being expected to provide guidance on how gender diverse people should be treated	Staff training and development
UK_9	Pansexual woman	24	Restaurant chef	catering	Casual homophobic comments	Stereotyping of LGBTI staff members	Diversity of staff in the workplace
